# A Specific Inflammatory Profile Underlying Suicide Risk? Systematic Review of the Main Literature Findings

**DOI:** 10.3390/ijerph17072393

**Published:** 2020-04-01

**Authors:** Gianluca Serafini, Valentina Maria Parisi, Andrea Aguglia, Andrea Amerio, Gaia Sampogna, Andrea Fiorillo, Maurizio Pompili, Mario Amore

**Affiliations:** 1 Department of Neuroscience, Rehabilitation, Ophthalmology, Genetics, Maternal and Child Health, Section of Psychiatry, University of Genoa, 16132 Genoa, Italy; valentina.maria.parisi@gmail.com (V.M.P.); andrea.aguglia@unige.it (A.A.); andrea.amerio@unige.it (A.A.); mario.amore@unige.it (M.A.); 2 IRCCS Ospedale Policlinico San Martino, 16132 Genoa, Italy; 3 Mood Disorders Program, Tufts Medical Center, Boston, MA 02111, USA; 4 Department of Psychiatry, University of Campania ’Luigi Vanvitelli’, 80138 Naples, Italy; gaia.sampogna@gmail.com (G.S.); andrea.fiorillo@unicampania.it (A.F.); 5 Department of Neurosciences, Suicide Prevention Center, Sant’Andrea Hospital, University of Rome, 00189 Rome, Italy; maurizio.pompili@uniroma1.it

**Keywords:** suicidal behavior, inflammatory cytokines, major depressive disorder, immunological differences, immune pathways

## Abstract

Consistent evidence indicates the association between inflammatory markers and suicidal behavior. The burden related to immunological differences have been widely documented in both major affective disorders and suicidal behavior. Importantly, abnormally elevated pro-inflammatory cytokines levels have been reported to correlate with suicidal behavior but whether and to what extent specific inflammatory cytokines abnormalities may contribute to our understanding of the complex pathophysiology of suicide is unknown. The present manuscript aimed to systematically review the current literature about the role of pro-inflammatory cytokines in suicidal behavior. Most studies showed a link between abnormally higher interleukin (IL)-1β, IL-6, tumor necrosis factor (TNF)-α, transforming growth factor (TGF)-β1, vascular endothelial growth factor (VEGF), kynurenic acid (KYN), and lower IL-2, IL-4, and interferon (IFN)-γ levels in specific brain regions and suicidal behavior. Unfortunately, most studies are not able to exclude the exact contribution of major depressive disorder (MDD) as a mediator/moderator of the link between inflammatory cytokines abnormalities and suicidal behavior. The association between suicidal patients (both suicide attempters or those with suicidal ideation) and the altered immune system was documented by most studies, but this does not reflect the existence of a specific causal link. Additional studies are needed to clarify the immune pathways underlying suicidal behavior.

## 1. Introduction

Suicidal behavior, which is frequently linked to mood disorders, in particular major depression, is a fundamental public health concern associated with significant disability and psychosocial impairment worldwide [1,2,3]. Major depression may be considered one of the leading causes of suicide worldwide with a more than 20-fold increased risk and at least half of all completed suicides which are linked to depressive disorders [1]. Generally, suicidal behavior associated with major depression occurs predominantly in the elderly due to the emergence of psychotic symptoms [1].

Abnormalities of the immune system have been reported to significantly contribute to the pathophysiology of both major depression [4] and suicidal behavior [5,6] based on existing evidence. Inflammatory mediators and oxidative stress leading to excitotoxicity may play a critical role in the pathophysiology of either major depression and suicidal behavior [7]. 

Studies in the current literature supported the abnormal activation of cell-mediated immunity and different immune biomarkers in the pathophysiological pathways underlying major affective disorders and suicidal behavior [4,5,6,7]. Specifically, abnormally elevated granulocytes and monocytes numbers, enhanced C-reactive protein (CRP) and haptoglobin, chemokines, abnormalities of regulatory T-cells, and inflammatory cytokines have been documented in both patients with major depression and suicidal behaviors. Thus, central and peripheral immune dysregulation are proposed as important pathways underpinning the pathophysiology of major depression and suicidal behavior identifying peripheral inflammatory mediators as promising candidate immune biomarkers.

In the last few decades, a growing interest emerged for inflammatory cytokines, which are known as key modulators of neuroinflammation, representing a heterogeneous group of molecules released by immunocompetent cells, such as lymphocytes and macrophages. Inflammatory cytokines include a heterogeneous group of messenger molecules released by immunocompetent cells playing a key role in stress-related conditions and major depression. One of the most relevant characteristics of inflammatory cytokines is the pleiotropy through which cytokines may bind to different cell target types. These molecules are generally divided into two subgroups: pro-inflammatory, including interleukin (IL)-1β, IL-6, interferon (IFN)-γ, and tumor necrosis factor (TNF)-α, and anti-inflammatory cytokines (e.g., IL-10, transforming growth factor-beta (TGF-β)).

Several studies recently postulated the existence of abnormally elevated levels of inflammatory cytokines in subjects at risk for suicide or those who die by suicide. 

For instance, Lindqvist and colleagues [8] reported increased levels of IL-6 in the cerebrospinal fluid of individuals who attempted suicide when compared to controls. Janelidze et al. [9] found abnormally elevated IL-6 and TNF-α concentrations together with reduced IL-2 levels in suicide attempters suggesting that cytokine levels in the blood may distinguish suicide attempters from depressed patients. Moreover, suicide attempters reported higher peripheral levels of both inflammatory cytokines and CRP, that is a pentameric protein in blood plasma, the circulating levels of which are higher in response to inflammation than depressed individuals who did not attempt suicide, independently of the time interval between suicidal behavior and cytokine quantification [5]. Relevantly, existing meta-analyses documented that circulating levels of IL-1β and IL-6 are abnormally elevated and IL-2 concentrations are significantly reduced in suicidal patients relative to non-suicidal patients and healthy controls promoting the hypothesis of inflammatory markers in suicidal patients (including those with active suicidal ideation as well as those with a positive history of suicide attempts) [6,10]. These studies supported the assumption that determining the inflammatory marker profile of suicidal patients may be crucial in order to better predict suicidal behaviors and identify novel therapeutic strategies.

In their postmortem study, Shelton et al. [11] investigated the expression of inflammatory genes in the Brodmann area of 10 depressed patients and found that genes coding for IL-1α and IL-2 were upregulated in the prefrontal cortex of depressed subjects. Furthermore, Pandey et al. [12] found that both the protein and messenger ribonucleic acid (mRNA) levels of IL-1β, IL-6, and TNF-α were abnormally altered in the prefrontal cortex of young adults who died by suicide, when compared to controls. Therefore, the available evidence supports the notion of existing abnormalities in pro-inflammatory cytokines in individuals who die by suicide as well as suicide attempters. 

Environmental, interpersonal stressors (e.g., social and familial threats, negative life events, etc.) may be triggering conditions underlying most suicidal acts. These precipitating/contributing factors may significantly interact with predisposing factors increasing vulnerability to suicide according to a stress-diathesis perspective [13], although the main pathophysiological mechanisms underlying this link are still poorly understood.

Such stressful conditions can activate inflammatory signaling pathways (e.g., nuclear factor kappa-lightchain-enhancer of activated B cell DeoxyriboNucleic Acid (NF-kB DNA) binding) in specific immune cells such as monocytes and macrophages linked to abnormally enhanced pro-inflammatory IL-1β, IL-6, and TNF-α levels [14,15,16,17,18,19,20] on neuroglial cells. Classically, neuroglial cells include astrocytes, oligodendrocytes, microglia cells and NG2 glia. In response to inflammatory and injury stimuli, microglia may develop different phenotypes and, when abnormally activated, microglia usually releases pro-inflammatory cytokines or chemokines such as IL-1, IL-6, IL-12, and TNF-α [21]. Pro-inflammatory cytokines released by microglia may stimulate astrocytes having phagocytic properties and secreting abnormally elevated cytokines such as IL-6 and TNF-α [22]. Finally oligodendroglia, commonly derived by oligodendrocytes progenitor cells, may release specific cytokines (e.g., IL-1) and chemokines (e.g., monocytes chemoattractant protein 1) playing a fundamental role in myelination [23]. Importantly, abnormally elevated inflammatory cytokines levels are able to determine a cytotoxic action on neuroglial cells, presumably leading to apoptosis and demyelination with the final result of increased glutamate release impairments in neuroplasticity mechanisms and enhanced excitotoxicity [7]. Given the mentioned background, the present manuscript aimed to systematically review the current literature about the role of pro-inflammatory cytokines in suicidal behavior.

## 2. Methods

### 2.1. Search Strategy and Study Selection

**Figure 1:**
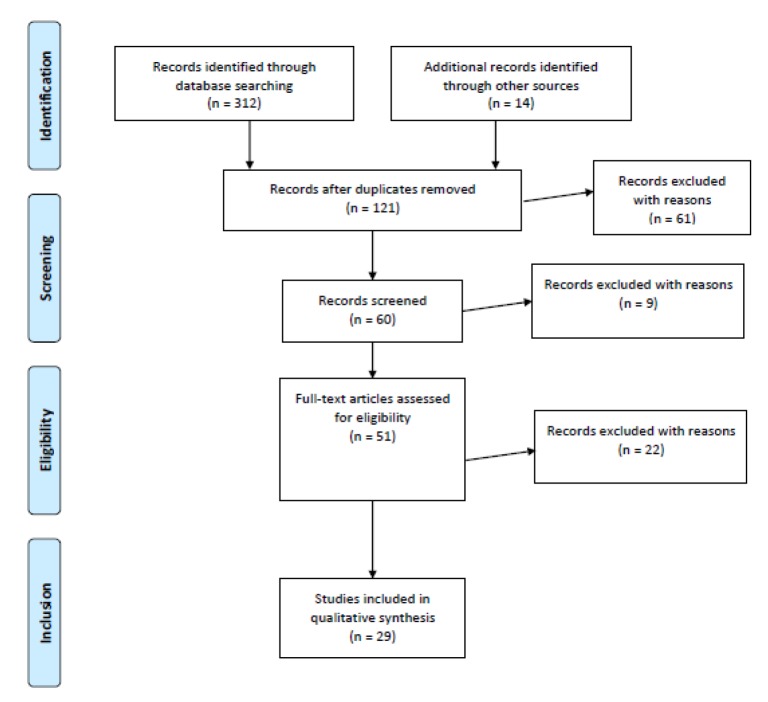
Study selection flowchart.

Relevant studies about the main topic have been identified using a detailed search strategy reported in Figure 1. Aiming to provide a systematic and critical review on inflammatory cytokines and their role in suicidal behavior, a detailed Pubmed/Medline, Scopus, Science Direct, and PsycInfo search has been conducted. All relevant studies within the time interval between 1990 and June 2019 (exclusively in English language) upon this topic have been searched. The search used a combination of the following terms: “Neuroinflammation” AND “Inflammatory Cytokines” OR “pro-inflammatory cytokines” OR “anti-inflammatory cytokines” AND “Suicid*” (including suicide ideation OR suicidal thoughts OR deliberate self-harm OR suicide attempts OR completed suicide). We examined all the full-text articles on the main topic in case a title or abstract seemed to describe a study eligible for inclusion. Two independent researchers (VMP, GS) carried out a two-step literature search. Consultations with senior authors (AF, MP, MA) have been used in case of any discrepancies between the two reviewers who, blind to each other, examined the studies for their possible inclusion. Relevant studies were also searched using the reference lists of the articles included in the review (VMP, GS). We included all English language full-text articles reporting original data about the main topic. The main search strategy and criteria for study selection (identification, screening, eligibility, inclusion process) in the current review are reported in Figure 1.

### 2.2. Study Design and Eligibility Criteria

In order to obtain a high standard of reporting, we adopted the ‘Preferred Reporting Items for Systematic Reviews and Meta-Analyses’ (PRISMA) guidelines [24]. PRISMA may be considered an evidence-based minimum set of items that is frequently used in both systematic reviews and meta-analyses. PRISMA usually allows the reporting of reviews assessing randomized clinical trials, but it may be similarly adopted for reporting systematic reviews of other research, such as reports referring to evaluations of interventions. The PRISMA statement includes a 27-item checklist and a four-phase flow diagram for reporting in systematic reviews and meta-analyses. PRISMA is usually adopted to include a reliable report of different types of health studies and it mainly aims to improve the quality of research used in decision-making in healthcare.

### 2.3. Selection Criteria

Inclusion criteria were papers reporting a link between inflammatory cytokines and suicidal behavior in both depressed and nondepressed individuals. Whether a title/abstract appears to refer to a study eligible for inclusion, the full-text article was down-loaded and carefully assessed to ascertain its possible relevance to the main topic according to inclusion/exclusion criteria. The following criteria have been used for the aims of this study: (a) being an original paper in a peer-reviewed journal and (b) describing the experimental association between (pro- or anti-) inflammatory cytokines and suicidal behavior. Exclusion criteria were: manuscripts referring exclusively to suicide attempts/suicidal ideation, articles with samples including individuals <18 years of age, articles that did not mention how the diagnosis has been performed, meta-analyses, main texts without abstracts, manuscripts that focused only on completed suicides, and those reporting only incomplete laboratory data.

### 2.4. Recorded Variables

The recorded variables for each article about inflammatory cytokines and suicide were: author(s), year, sample features, study design, type of inflammatory cytokines which have been investigated, main results, major shortcomings, and conclusive remarks (Table 1 and Table 2).

Although we did not include a risk of bias assessment, according to the PRISMA statement the following criteria have been considered in order to detect the fundamental limitations potentially affecting the validity and generalization of the included studies: (a) representativeness of the main study sample; (b) existence/representativeness of a control group; (c) study design; (d) existence of a long-term follow-up period; (e) evidence-based measures to assess suicide risk; and (f) existence of at least two raters who blind to each other identified the most relevant studies for inclusion.

## 3. Results

### 3.1. Number of Selected Studies

Initially, a total of 312 full-text articles (149 from Pubmed, 46 from Scopus and 117 from ScienceDirect, and PsycInfo) emerged using the combined search strategy; after a thoroughly analysis, 60 full-text articles have been screened and 191 completely excluded after removing duplicates. Later, 61 additional full-text articles were further deleted as they were: (1) articles not published in peer reviewed journals, (2) articles not in English language, and (3) articles without abstracts. In addition, nine further articles were subsequently excluded as: (1) their abstracts did not specify in detail the association between inflammatory cytokines and suicidal behavior, (2) they were published before 1990, (3) include unclear information about materials and methods or number of recruited patients. Finally, of the fifty-one articles which were evaluated for eligibility, 22 additional full-texts were excluded due to the low-relevance to the main topic. Therefore, the final result consists of 29 studies (including 1525 patients and 1400 controls) that fulfilled inclusion criteria.

### 3.2. Studies Directly Analyzing Inflammatory Abnormalities in Suicidal (Completed Suicides or Those with a History of Suicide Attempts) Patients

Overall, 11 studies analyzed the existence of inflammatory cytokines abnormalities in suicidal *vs.* nonsuicidal healthy controls. Recently, Conejero et al. [25] reported that IL-1β was negatively associated with right orbitofrontal cortex (OFC) activation during the explicit social exclusion *vs.* social inclusion, whereas IL-2 was positively associated with activation of the right anterior cingulate cortex (ACC), insula and OFC even after controlling independently for the suicidal status. Moreover, Pandey et al. [4] found that IL-1β, IL-6, TNF-α, and lymphotoxin A mRNA and protein levels were significantly increased, while IL-10 and IL-1 receptor antagonists (IL-1ra) were reduced in the prefrontal cortex (PFC) of depressed individuals who died by suicide compared to healthy controls. The same research group [26] previously documented that both protein and gene expression of glucocorticoid receptor-α (GR-α) were reduced in the PFC and amygdala of teenage suicide victims (*n* = 24) compared to healthy controls. 

The GR inducible target gene glucocorticoid-induced leucine zipper (GILZ) mRNA levels were decreased in PFC and amygdaloidal nuclei of these subjects as well. Based on another recent study [27], TNF-α expression was significantly higher in the dorsolateral prefrontal cortex (dlPFC) of suicide subjects regardless of psychiatric diagnosis, although its expression was enhanced even in major depressive disorder (MDD) subjects who died by causes other than suicide. Moreover, in a two-year follow-up study, Bay-Richter et al. [28] demonstrated that quinolinic acid (QA) was increased and kynurenic acid (KYN) decreased in suicidal patients *vs.* healthy controls. A significant association between lower KYN and severe depressive symptoms and IL-6 levels and more severe suicidal symptoms were also documented. Another additional study [9] analyzed the levels of specific inflammatory markers in suicidal (*n* = 47), non-suicidal depressed patients (*n* = 17) and healthy controls (*n* = 16) and found greater IL-6 and TNF-α levels coupled with decreased IL-2 concentrations in the group of suicide attempters after adjusting for potential confounders. 

Moreover, Sublette et al. [29] found that KYN was higher in the MDD suicide attempter subgroup compared with MDD non-attempters who did not differ from healthy controls. Only suicide attempters showed a positive correlation of the cytokine activation marker neopterin with the KYN: tryptophan (TRP) ratio, concluding that the production of KYN may be influenced by inflammatory processes. Cerebrospnal fluid (CSF) levels of IL-6 were also found to be higher in suicide attempters than healthy controls [8], with subjects having a history of violent suicide attempts showing the highest levels of IL-6. A significant positive correlation between Montgomery–Åsberg Depression Rating Scale (MADRS) scores and CSF IL-6 levels were reported. Importantly, CSF IL-6 and TNF-α correlated with 5-hydroxyindolacetic acid (5-HIAA) and homovanillic acid (HVA). Significantly reduced plasmatic TNF-α concentrations compared to non-suicidal MDD adolescents (*n* = 18) were also reported by Gabbay et al. [30] (2009) in a sample of 12 suicidal MDD adolescents. Even after controlling for age and gender, IFN-γ was increased in both suicidal MDD and non-suicidal adolescents compared to controls. Additionally, in the study of Kim et al. [31], non-suicidal MDD patients (*n* = 33) had higher IL-6 production than suicidal MDD patients (*n* = 36) and healthy controls (*n* = 40). Suicidal MDD patients had lower IL-2 compared to non-suicidal patients and normal controls as well, while both MDD patients with and without a history of suicide attempts had lower levels of IFN-γ and IL-4 and a higher TGF-β1 production. Finally, Lee and Kim [32] found that in vitro TGF-β1 levels resulted significantly higher in suicidal MDD patients (*n* = 48) and non-suicidal MDD patients (*n* = 47) than controls (*n* = 91).

### 3.3. Studies Analyzing Inflammatory Abnormalities in Suicidal Patients (with a Positive History of Suicide Attempts and Suicidal Ideation)

Four studies analyzed the existence of inflammatory cytokines abnormalities in suicidal patients (having both a positive history of suicide attempts and suicidal ideation) and eventually healthy controls. First, Knowles et al. [33] tried to identify a possible link between increased inflammatory cytokines and suicide risk and reported that IL-8 and IL-6 shared a significant genetic overlap with risk of suicide attempts (for IL-6 this risk was attenuated when body mass index (BMI) was included as a covariate). Relevantly, the genetic overlap between IL-8 and risk for suicide attempts was significant in females but not males. According to Keaton et al. [34], the biological profile of patients considered to be at increased risk of suicide differed from that of depressed individuals. Importantly, blood cell count and polymorphonuclear leukocyte count had a significant impact on suicide risk. The authors reported that IL-8 was negatively associated with increased suicide risk after adjusting for confounders. Furthermore, Melhem et al. [35] found lower hair cortisol concentrations in first-time suicide attempters compared to patients with suicidal ideation and normal controls. 

Moreover, patients with suicide attempts showed lower GR or the DNA methylation of human glucocorticoid receptor gene (NR3C1) (α isoform) mRNA, higher CRP, and higher TNF-α mRNA supposing that suicide attempters demonstrated a distinct biological profile on the identified biomarkers. Finally, it has been already suggested that T-cells of depressed suicidal depressed patients may have T-helper1 (Th1) characteristics, while T-cells of non-suicidal depressed patients may have Th2 characteristics [36] based on the concept that Th1 activation in suicidal depression may reflect, similarly to other autoimmune disorders (e.g., autoimmune diabetes, multiple sclerosis, and autoimmune thyroiditis), a specific form of self destructive activation of the immune system.

### 3.4. Studies Exploring Inflammatory Abnormalities in Depressed Patients with Suicidal Ideation vs. Those Without

Two studies explored the existence of inflammatory cytokines abnormalities in depressed patients with suicidal ideation *vs.* those without. First, O’Donovan et al. [37] reported that MDD patients with suicidal ideation had higher inflammatory index scores than both controls and MDD patients with lower suicidal ideation. MDD patients also had higher levels of IL-6 and IL-10 than normal controls at the follow-up analyses. A trend toward higher levels of CRP in MDD patients was reported as well. Additionally, patients with suicidal ideation had higher levels of IL-6 and a trend toward lower levels of IL-10 than healthy controls. In addition, thirty female outpatients with recurrent MDD (18 with suicidal ideation and 12 without) together with 16 healthy controls were also analyzed in the study of Grassi-Oliveira et al. [38]. After multivariable analysis of covariance adjusted for age, BMI, and depression severity, the authors documented that MDD patients with suicidal ideation presented lower levels of monocyte chemoattractant protein-1/chemokine C-C motif ligand 2 (MCP-1/CCL2) and normal T-cell expressed and secreted (RANTES/C-C motif ligand 5 CCL5 or RANTES/CCL5) and higher levels of Eotaxin/C-C motif chemokine 11 (CCL11) when compared to healthy controls. 

### 3.5. Studies Investigating Inflammatory Abnormalities in Patients with a History of Suicide Attempts vs. Those Without

Seven studies investigated the existence of inflammatory cytokines abnormalities in patients with a history of suicide attempts *vs.* those without. Coryell et al. [39], in a sample of MDD patients with a history of suicide attempts (*n* = 79), reported that IL-1β levels were lower when compared with those of subjects without a history of suicide attempts (*n* = 123). Moreover, IL-1β levels correlated inversely with aggression measures. Kim et al. (2013) reported that the GG genotype of the TNF-α-308G>A polymorphism increased the risk for suicide attempts. In addition, IFN-γ +874A>T and IL-10 -1082A>G were not associated with suicide risk. In the longitudinal study of Janelidze et al. [40], CSF eotaxin-1, macrophage inflammatory protein-1β (MIP-1β), monocyte chemoattractant protein-1 (MCP-1), monocyte chemoattractant protein-4 (MCP-4), and thymus and activation-regulated chemokine (TARC) were lower in the group of suicide attempters than healthy controls. Moreover, lower chemokine levels were associated with psychotic symptoms and pain. After follow-up analyses, TARC was significantly lower in suicide attempters compared to psychiatric patients who had never attempted suicide. A positive correlation between blood TARC and brain-derived neurotrophic factor (BDNF) levels emerged. In 2013, Vargas et al. [41] reported that subjects with a history of suicide attempts (*n* = 141) had significantly higher levels of nitrogen oxide (Nox) and lipid hydroperoxides and lowered plasma total antioxidant potential (TRAP) when compared to individuals without suicide attempts (*n* = 201). After logistic regression analysis, both unipolar and bipolar disorder, female gender, smoking behavior, and lipid hydroperoxides were associated with a history of suicide attempts independently of specific socio-demographic and clinical risk factors. 

Isung et al. [42] aimed to identify new inflammatory biomarkers for suicide prediction and found lower levels of vascular endothelial growth factor (VEGF) in the seven patients who completed suicide after a follow-up period of 13 years. VEGF also showed a trend for negative correlation with the planning subscale of the Suicide Intent Scale. A trend emerged for lower IL-2 and higher IFN-γ levels in suicide victims as well. The same research group [43] found the existence of a significant negative correlation between CSF VEGF and depression severity in a cross-sectional study, including medication-free suicide attempters and 20 healthy male volunteers. The authors hypothesized that lower CSF levels of VEGF may reflect a lack of trophic support to neurons and downregulation of neurogenesis in the hippocampus associated with more severe depressive states. Finally, in 1993 Nassberger and Träskman-Bendz [44] had already identified an association between S-IL-2R and the ratio of norepinephrine-epinephrine in 24-h urine together with plasma and cerebrospinal fluid 4-hydroxy-3-methoxymethylglycol in suicide attempters. 

### 3.6. Studies Exploring Cytokines Abnormalities in Suicide Completers vs. Subjects Who Died for Other Causes

Three studies explored cytokines abnormalities in patients who died by suicide *vs.* those who died for other causes. Boehm et al. [45] analyzed the lungs of burn victims and showed a greater extent of intra-alveolar edema than the other groups. The authors added that macrophages in all groups mostly showed a distinct expression of TNF-α, but not of IL-8 or intercellular adhesion molecule-1 (ICAM-1), that the intravascular erythrocytes positivity of TNF-α was strongest in the group of burn victims. Tonelli et al. [46] reported an elevated expression of IL-4 in female suicide victims and IL-13 in male suicide victims. Abnormally elevated, although not significant, cytokine expression was also observed for TNF-α in female suicide victims. Finally, Torres-Platas et al. [47] reported that blood vessels surrounded by a high density of macrophages were more than twice higher in depressed suicides than healthy controls. Moreover, gene expression of ionized calcium-binding adapter molecule 1 (IBA1) and MCP-1 was significantly upregulated in depressed suicides, and mRNA for CD45 was significantly enhanced in depressed suicides.

### 3.7. Studies Investigating Inflammatory Abnormalities in Depressed Patients Who May be Indirectly at Risk for Suicide

Two studies reported the existence of inflammatory cytokines abnormalities in depressed, suicidal patients with suicidality *vs.* healthy individuals. IL-6 was significantly elevated in melancholic depressive patients (*n* = 29) compared to healthy controls (*n* = 39), while no differences were found between patients with atypical depression (*n* = 18) and healthy volunteers (*n* = 39) [48]. Lower TNF-α serum level was found both in melancholic and atypical depressed individuals compared to healthy subjects. A positive correlation between cytokine levels and atypical depression was also found. Importantly, the duration of lifetime exposure to antidepressants correlated with IL-6 serum levels in both melancholic and atypical depressed subjects. In addition, Li and colleagues [49] reported that plasma TNF-α levels were significantly decreased following venlafaxine treatment in a sample of 64 first-episode drug-naïve MDD patients when compared with 64 matched healthy controls. Relative to non-responders, responders had a greater reduction in TNF-α levels, which was associated with a greater reduction rate of depressive symptoms. The plasma TNF-α levels were equally higher in both suicidal and non-suicidal MDD patients compared to healthy controls at admission.

## 4. Discussion

### Summary of Main Findings and Review of Study Designs

According to the main findings of this systematic review, neuroinflammation may play a relevant role in the pathophysiology of suicidal behavior. However, based on the selected studies and in line with a previously published paper of our research group upon the same topic [7], the existence of an association between inflammatory cytokines abnormalities and suicidal behavior does not necessarily reflect a specific causal link. Among the selected studies, eleven reports [8,9,12,25,26,27,28,29,30,31,32] analyzed inflammatory cytokines abnormalities in suicidal patients, four [33,34,35,36] explored cytokines abnormalities in patients with both a positive history of suicide attempts and suicidal ideation, two [37,38] investigated the existence of inflammatory cytokines abnormalities levels in depressed patients with suicidal ideation *vs.* those abnormally without, seven [31,40,41,42,43,44,50] examined the inflammatory cytokines abnormalities in patients with a history of suicide attempts *vs.* those without, three [45,46,47] analyzed patients died by suicide *vs.* those died for other causes, and two [48,49] focused on depressed suicidal patients with suicidality.

There are studies showing the existence of a distinct biological profile underlying suicidal behavior with inflammatory dysregulation that may be significantly involved in the physiopathology of mood disorders with suicidality [51,52,53]. Overall, the existence of an imbalance among pro-inflammatory (e.g., IL-1β, IL-2, IL-6, IFN-γ, and TNF-α) [54,55,56] and anti-inflammatory cytokines (e.g., IL-4 and IL-10) has been reported even in untreated depressed patients.

Systematic reviews and meta-analyses [6,10,52] confirmed the relation between circulating inflammatory markers, major affective disorders, and suicidal behavior with significant inflammatory changes observed in the periphery, CSF, and brain tissues of suicidal patients. 

In particular, the meta-analysis of Enache et al. [52] supported the existence of abnormally increased IL-6 and TNF-α concentrations in CSF and brain parenchyma, together with an increased microglia activity and abnormally reduced astrocytes/oligodendrocytes markers levels in MDD. The authors supposed that the reduced number of astrocytes can induce impairments in the integrity of blood-brain barrier with enhanced monocyte recruitment and inflammatory infiltration, which has been confirmed by post-mortem studies in MDD populations.

What are the neurobiological mechanisms underlying the link between abnormally inflammatory cytokines levels and suicidal behavior? The first demonstration of a causal link between inflammation and subsequent onset of depression comes from the initial treatment trials using IFN in patients with specific infections or IFN-β in multiple sclerosis with depressive symptoms and suicidal behavior; according to these studies, depressive symptoms may emerge approximately one month after the first administration of this medication [57,58].

Based on most recent evidence, neuroinflammation may determine abnormalities in the kynurenine pathway, resulting in increased quinolinic acid production, which agonizes N-methyl-D-aspartate (NMDA) receptors resulting in neurotoxic effects potentially contributing to the pathophysiology of both major affective disorders and suicidal behavior [29,59,60,61]. Specifically, inflammatory cytokines are able to activate the indolamine 2,3-dioxygenase (IDO), a specific enzyme which is located on microglia and astrocytes. IDO is implicated in the catabolism of TRP, which is involved in serotonin neurotransmission. Kynurenine pathway usually produces kynurenic and quinolinic acid, which are highly neurotoxic metabolites able to activate NMDA receptors [62]. An altered production of both kynurenic and quinolinic acid are supposed to be related to a severe unbalance between microglia and astrocytes activation [63]. 

According to our findings, the most consistent link between inflammatory changes and suicidal behavior may be found in studies directly analyzing cytokines abnormalities and suicidal patients (completed suicides or those with a history of suicide attempts) when compared to healthy controls. Most of these studies documented abnormally higher levels of IL-1β, IL-6, TNF-α, TGF-β1, VEGF, and KYN, and lower levels of IL-2, IL-4, and IFN-γ predominantly in specific brain regions such as OFC, right ACC, insula, and dlPFC of suicidal patients (mainly suicide attempters) [8,9,12,25,26,27,28,29,30,31,32,39,42,43]. Abnormally elevated IL-6 and IL-10 levels have also been reported in patients with active suicidal ideation when compared to healthy controls [37]. 

Based on functional neuroimaging studies, social exclusion may pathologically activate brain regions like the insula, OFC, and ACC [64] playing a fundamental role in suicidal vulnerability [65,66,67], which may be enhanced by neuroinflammation [14]. In addition, inflammatory cytokines may lead to significant behavioral and emotional changes according to their direct effects in specific brain regions [53]. However, other studies support the notion that the inflammatory modulation of the neural response to social exclusion may be directly related to specific psychiatric conditions, independently of suicidal behavior [25]. To this specific regard, the authors found that IL-1β was negatively associated with right OFC activation in explicit social exclusion *vs.* social inclusion, whereas IL-2 was positively associated with activation of the right ACC insula and OFC in explicit social exclusion *vs.* social inclusion, even after controlling for group, indicating that these changes were independent of suicidal status.

Not all studies supported the association between abnormally elevated cytokines concentrations and suicidal behaviors. For instance, the finding of abnormally reduced TNF-α in suicidal depressed young adults compared to non-suicidal controls is not in line with what most adult studies usually indicate. According to a systematic review on this topic, this can be due to the existence of different underlying pathophysiology concerning suicidal behaviors in adolescents and young adults [68]. Increased suicide risk has also been found in children and adolescents treated with antidepressant medications, which should be closely monitored, especially in the initial treatment phases [69]. The increased suicidal risk in this patient population may be related to initially induced antidepressants effects in youths such as enhanced activation, hyperarousal associated with impulsivity, restlessness, and/or insomnia [70].

We firmly suggest that further additional studies using longitudinal designs should test the association between abnormal inflammatory response and suicidal behaviors in homogeneous populations after controlling for the presence of major depression.

This study needs to be considered according to the following shortcomings. First, the majority of studies included in the present review (76.7%) adopted a cross-sectional study design and lack of appropriate longitudinal follow-up periods. In addition, patients with different diagnostic subtypes (e.g., melancholic, atypical, etc.) and heterogeneous treatment and disease duration have been selected. Moreover, the selection of mixed samples of patients (e.g., patients with different psychiatric diagnoses or comorbid psychiatric conditions) does not permit the generalization of the main results of the selected studies. In addition, some studies lack control groups or healthy controls may not be appropriately matched for socio-demographic and clinical characteristics. Furthermore, both psychiatric symptoms and suicidal behavior may have been evaluated only clinically and not using specific psychometric measures. Most studies (e.g., postmortem studies) did explore the effects of specific inflammatory cytokines on some brain regions (such as OFC, ACC, and PFC) rather than others; the interaction with other significant neurobiological variables may not have been taken into account as well. Importantly, the short- and long-term immunoregulatory effects of psychoactive treatments taken by patients may represent additional confounding factors in specific studies. Furthermore, although conducted on the most recent evidence-based contributions in the current literature, the reviewed studies are based on the authors’ choice and do not necessarily represent the most representative studies upon the main topic. Notably, we were not able to include within the main text a risk of bias assessment that would have helped us to establish transparency of evidence synthesis results and findings for each of the included studies.

Finally, most studies could not exclude the exact contribution of MDD as a mediator/moderator of the link between inflammatory cytokines abnormalities and suicidal behavior. Inflammatory changes have been reported to occur with great variability not only in MDD, but also in other psychiatric conditions such as schizophrenia and related psychoses in which the role of the immune abnormalities has been consistently reported [71]. To this specific regard, schizophrenia has been linked to the disruption of the cytokine milieu and the enhanced propensity for the production of specific proinflammatory cytokines. In addition, given the most frequent cross-sectional nature of the studies’ designs, it is not possible to exactly determine whether inflammation precedes depression/suicide or vice-versa. Thus, how and to what extent inflammatory cytokines abnormalities are related to MDD or other comorbid psychiatric conditions and not exclusively related to suicidal behavior is not quite unclear.

## 5. Conclusions

Based on the most relevant results of the present review and in line with previous findings, neuroinflammation may play a crucial role in the pathophysiology of suicidal behavior [7,72]. Specific inflammatory changes may be identified in the periphery, CSF and central nervous system of patients at risk for suicide. More detailed knowledge of the pathophysiological mechanisms underlying suicidal behavior, including possible mediators/moderators of the inflammatory response that are able to enhance vulnerability or resilience to suicide is really desirable for both clinicians and researchers. Importantly, it would be appreciated to identify specific subgroups of suicidal patients who also manifest an increased vulnerability to inflammation in order to more directly detect specific at-risk subjects as well as target homogeneous populations to test the precision of possible anti-inflammatory agents.

Currently, we are only at the beginning of this exciting journey that might lead clinicians in the next future to use immunomodulatory treatments alone or in combination with existent available psychotropic medications in order to manage psychiatric conditions such as major affective disorders and suicidality. Further additional studies are required in order to clarify the complex pathophysiological mechanisms of the immune pathways underlying suicidal behavior.

## Figures and Tables

**Table 1:** Most relevant cross-sectional studies showing the association between inflammatory cytokines and suicidal behavior.

Author (s), Year	Sample Characteristics	Study Design	Inflammation Measurement	Main Findings	Limitations	Conclusions
Conejerob et al. (2019) [25]	42 SA, 40 affective controls, 19 healthy controls	Cross-sectional case-control study	IL-1β IL-6 TNF-α IL-2	IL-1β was negatively associated with right orbitofrontal cortex activation in ESE vs. INC, whereas IL-2 was positively associated with activation of the right anterior cingulate cortex, insula, and orbitofrontal cortex in ESE vs. INC.	1. Medicated patients reflecting real-life conditions were recruited; 2. only females were included; 3. results for smoking status and BMI were not controlled; 4. the healthy controls group was smaller than the affective controls and SA groups.	Baseline IL-1β and IL-2 blood levels are differentially associated with cerebral activation involved in the perception of social exclusion, independently of suicidal behavior.
Wang et al. (2019) [27]	16 non-psychiatric controls and 43 suicide subjects (21 MDD-suicides and 22 suicides with other psychiatric disorders)	Cross-sectional case-control study	TNF-α	TNF-α expression was significantly higher in dlPFC of suicide subjects regardless of psychiatric diagnosis. Its expression level was also increased in MDD subjects who died by causes other than suicide. Conversely, the expression of miR-19a-3p was upregulated in suicide subjects.	1. The power of the study is quite low; 2. the study lacks depressed patients without suicidal ideation.	This study provides mechanistic insights into the dysregulation of TNF-α gene in suicide brain, which could potentially be involved in suicidal behavior.
Keaton et al. (2019) [34]	66 females with mood and anxiety disorders	Cross-sectional study	IL-6 IL-8	Increased IL-6, lymphocytes, monocytes, white blood cell count, and polymorphonuclear leukocyte count levels significantly impacted suicide risk (the latter two inferring the strongest influence). IL-8 was independently and negatively associated with enhanced suicide risk, even after adjusting for confounders.	1. The cross-sectional study design; 2. the findings may only identify associations and are not able to prove causality.	The biological profile of patients assessed to be at increased suicide risk differed from that associated with depression.
Knowles et al. (2019) [33]	1882 subjects of which 159 with SA and 135 with SI	Cross-sectional study	IL-6 IL-8	IL-8 and IL-6 shared significant genetic overlap with risk for SA (for IL-6 this was attenuated when BMI was included as a covariate). The genetic overlap between IL-8 and SA risk was significant only in females but not males.	1. Personality traits could confound or mediate the main results; 2. the results may be population (Mexican American) specific; 3. increased circulating cytokine levels were observed in response to stress; 4. environmental influences might have increased inflammation.	Cytokine abnormalities are not a secondary manifestation of suicidal behavior, but play a fundamental role in the pathophysiology of suicide attempts.
Coryell et al. (2018) [39]	Patients in major depressive episodes who had a history of two or more SA (*n* = 79), or no history of SA (*n* = 123)	Cross-sectional study	CRP IL-6 IL-1β IL-1ra TNF-α.	One of five of the inflammatory markers (IL-1β), distinguished the two groups with lower values in the SA group. IL-1β levels correlated inversely with measures of aggression but neither impulsivity or aggressive behavior appear to explain the association between IL-1β levels and SA status.	1. Small sample sizes; 2. patients described here were not treatment naïve; 3. antidepressant treatment may or may not have influenced cytokine levels.	Results identify recent aggressive behavior, higher levels of impulsivity, and lower levels of IL-1β as risk factors for a history of multiple SA in a group with major depressive episodes. These measures appear to be additive in their effects.
Melhem et al. (2018) [35]	38 with SA, 40 with SI, 37 healthy controls	Cross-sectional case-control study	HCC	Lower HCC [β = −0.55, 95% CI (−0.96, −0.13), *p* = 0.01, ES = −0.54] were found in those with SA for the first time compared to those with SI and controls.	1. It is not clear whether HPA axis dysregulation exists prior to suicidal behavior or as a consequence of an attempt because existing studies examine cortisol levels in subjects with history of suicidal behaviour; 2. small sample size; 3. cross-sectional study design.	This is the first study to differentiate youths who attempt suicide from those with SI on HCC. The present study also showed that low HCC precedes SA.
Pandey et al. (2018) [4]	24 depressed individuals who died by suicide and 24 non-psychiatric controls	Cross-sectional case-control study	IL-1β, IL-6, TNF-α, Lymphotoxin A, Lymphotoxin B, IL-8, IL-10, IL-13	Protein levels of IL-1β, IL-6, TNF-α, and lymphotoxin a were significantly increased, and levels of anti-inflammatory cytokine IL-10 and of IL-1ra were significantly reduced in the prefrontal cortex of depressed individuals who died by suicide compared with controls.	1. Some of the suicide group had been taking antidepressant medications at the time of death.	Alterations of cytokines may be associated with the pathophysiology of depressed suicide and there may be an imbalance between pro- and anti-inflammatory cytokines in subjects who died by suicide.
Torres-Platas et al. (2015) [47]	24 depressed suicides and 17 controls (without psychiatric, neurological or inflammatory illnesses)	Cross-sectional study	Gene expression of IBA1 and MCP-1	Gene expression of IBA1 and MCP-1 was upregulated in depressed suicides. In addition, mRNA for CD45 was also increased in depressed suicides. An increase compared to controls was found in the proportion of blood vessels surrounded by a high density of CD45-IR cells (non-significant difference).	1. Brains from depressed suicides may be more highly exposed to peripheral cytokines crossing the blood-brain barrier; 2. other types of macrophages (including microglia), together with perivascular macrophages, may account for the observed increase in IBA1-IR cells associated with blood vessels in case samples.	Depression- and suicide-associated increases in circulating pro-inflammatory cytokines may be linked to low-grade cerebral neuroinflammation involving the recruitment of circulating monocytes.
Pandey et al. (2013) [26]	24 teenage suicide victims and 24 matched normal controls	Cross-sectional case-control study	IL-1β, IL-6, TNF-α	The mRNA and protein expression levels of IL-1β, IL-6, and TNF-α were significantly increased in Brodmann area 10 of suicide victims compared with normal controls.	1. Stress effects (with associated changes in GR-α) on the hippocampus cannot be observed in children or adolescents, but only in adults; 2. changes in the GR-α may be not related to suicidal behavior but may change as a result of early life trauma.	Increases of pro-inflammatory cytokines in the post-mortem brain of teenage suicide victims suggest that TNF-α, IL-1β, or IL-6 are associated with the neurobiology of suicide and that targeting these cytokines may help in developing new therapies for the treatment of suicidal behavior.
Kim et al., (2013) [50]	204 patients with SA and 97 control patients without SA	Cross-sectional case-control study	TNF-α, IL-10, IFN-γ	The GG genotype of the TNF-α −308G>A polymorphism significantly increased SA risk. IFN-γ +874A>T and IL-10 −1082A>G were not associated with risk for suicide. Lethality of the SA was not associated with any of the three cytokine genotypes or allele types.	1. The relatively small sample size; 2. cross-sectional study design	TNF-α −308G>A polymorphism may be considered an independent risk factor for SA in MDD.
Vargas et al. (2013) [41]	342 subjects divided into those with (*N* = 141) and without (*N* = 201) a history of SA	Cross-sectional case-control study	CRP Fibrinogen ESR IL-6 TNF-α	Subjects with SA had higher nitricoxide metabolites and lipid hydroperoxides levels and reduced plasma total antioxidant potential than those without. Regression analyses showed that unipolar/bipolar disorder, female gender, smoking behavior, and lipid hydroperoxides were linked to a history SA independently of classical risk factors.	1. Participants were recruited from the center for smoking cessation treatment; 2. inflammatory, oxidative/nitrosative stress and metabolic biomarkers were assessed at baseline and correlated with a history of SA (as trait and not state-markers); 3. results may only delineate associations and not causality	Oxidative stress, nitrosative stress, lowered antioxidant levels may play a role in the pathophysiology of suicidal behavior independently from the effects of depression and smoking and classical suicide predictors (e.g., years of education and marital status).
Dunjic-Kostic et al. (2013) [48]	29 melancholic, 18 atypical MDD patients, and 39 healthy controls	Cross-sectional case-control study	TNF-α, IL-6	IL-6 was significantly elevated in MDD-M. Lower TNF-α serum level was found both in melancholic patients and those with atypical depression. We detected a positive correlation between cytokine levels in atypical, but not in melancholic subjects. Clinical parameters (e.g., duration of illness, current episode, age of onset) were related to cytokine levels in atypical depression, while the duration of lifetime exposure to antidepressant treatment correlated to IL-6 serum levels in both patients with melancholic and atypical depression.	1. The cross-sectional study design; 2. the lack of information regarding the association between inflammatory markers and antidepressant treatment over time	The study showed certain differences in pro-inflammatory cytokine serum levels in melancholic and atypical depressed patients than healthy subjects. Importantly, IL-6 elevation might represent a state indicator for acute exacerbation, especially in melancholic patients.
Grassi-Oliveira et al. (2012) [38]	30 female outpatients with recurrent MDD divided in two groups according with the presence/ absence of SI, and 16 healthy controls	Cross-sectional case-control study	MCP-1, CCL2, CCL5, CCL11	MDD patients with suicidal ideation presented lower levels of MCP-1, CCL2 and CCL5 and higher levels of CCL11 compared to healthy controls. These differences remained significant after adjusting for depression severity.	1. Small sample size; 2. participants included in MDD groups were taking antidepressants; 3. nicotine users from the sample were not excluded; 4. only female participants were included.	Findings indicated that the presence of recurrent MDD with suicidal ideation is associated with differences in inflammatory chemokines when compared to those without suicidal ideation.
Isung et al. (2012b) [43]	43 medication-free suicide attempters and 20 healthy male controls	Cross-sectional study	CSF VEGF, CSF IL-8, and IL-6 levels measured with an ultra-sensitive immunoassay system	Suicide attempters showed lower CSF VEGF and IL-8 levels than healthy controls. Also, a significant negative correlation was observed between CSF VEGF and severity of depression. A more severe depressive state was correlated with low CSF levels of VEGF reflecting a lack of trophic support to neurons and down-regulation of hippocampal neurogenesis.	The study was cross-sectional in nature	IL-8 may be crucial in neuroprotection. A role for an impaired innate immunity and dysregulation of neuroprotection has been suggested in both depression and suicidal behavior.
Sublette et al. (2011) [29]	Fourteen subjects with MDD and a history of SA compared with 16 MDD patients without and 31 healthy controls	Cross-sectional case-control study	KYN, TRP, and the cytokine activation marker neopterin were investigated using high performance liquid chromatography	A priori planned contrasts showed that KYN was higher in the MDD SA subgroup compared with MDD non-attempters. KYN but not TRP was associated with attempt status, and only suicide attempters showed a positive correlation of the cytokine activation marker neopterin with the KYN:TRP ratio, suggesting that KYN production may be influenced by inflammatory processes among suicide attempters.	1. This study did not measure inflammatory indices apart from KYN, TRP, and neopterin levels; 2. the small sample size	These preliminary results suggest that KYN and related molecular pathways may be implicated in the pathophysiology of suicidal behavior. Our findings raise the possibility that pharmacologic manipulation of KYN levels might reduce suicide risk.
Janelidze et al. (2011) [9]	47 SA and 17 non-suicidal depressed patients 16 healthy controls.	Cross-sectional study	IL-2 IL-6 TNF-α	Increased levels of IL-6 and TNF-α as well as decreased IL-2 concentrations in SA were found compared to non-suicidal depressed patients and healthy controls.	1. Retrospective data, such as the duration of treatment and disease, were not registered; 2. data on smoking habits were not available; 3. the SA method, which was intoxication in 91% of patients in our sample; 4. storage time between sample collection and cytokine analysis was different for suicide attempters and depressed/healthy control subjects.	These results demonstrate for the first time that suicidal patients display a distinct peripheral blood cytokine profile compared to non-suicidal depressed patients.
Boehm et al. (2010) [45]	40 forensic autopsy cases of burn victims were examined 1 h after fire exposure and compared with 48 autopsy cases divided in post-mortem burns, deaths (e.g., hemorrhagic shock, railway suicide deaths)	Post-mortem study	TNF-α, IL-8, and ICAM-1 measured using immunohistochemical studies of lung tissue probes	Significantly higher extent of intra-alveolar edema was observed in the lungs of burn victims compared to other groups. A distinct expression of TNF-α, but not IL-8 or ICAM-1 was found in macrophages of all groups. A significantly stronger positivity of TNF-α in the group of burn victims was reported in intravascular erythrocytes when compared with other control groups.	1. The survival times (>1 h) of the cohorts may be too short to reach the phase of leukocyte immigration for further morphological changes (no reactive inflammatory cell infiltrates were found); 2. the small sample size limits the generalization of the present findings.	A non-specific immune response to fire-induced inhalation trauma was demonstrated by the positive reaction of TNF-α in erythrocytes of burn victims.
Lindqvist et al. (2009) [8]	63 SA divided in violent or non violent SA and 47 healthy controls	Cross-sectional case-control design	CSF and plasma IL-1β, IL-6, IL-8, TNF-α were measured. The relation between cytokines and monoamine metabolites, 5-HIAA, HVA, and MHPG in CSF was also evaluated	Suicide attempters showed significantly higher CSF IL-6 levels compared to healthy controls. Specifically, violent SA showed the highest IL-6. A significant positive correlation between MADRS scores and CSF IL-6 levels was found in all patients. CSF 5-HIAA and HVA were found to correlate with IL-6 and TNF-α but not with MHPG.	1. In order to test whether IL-6 is directly related to depressive and suicidal symptoms, cytokines should be administered peripherally or into the CNS; 2. the interaction between cytokines, monoamines, and HPA axis was not assessed.	CSF IL-6 plays a crucial role in suicidal behavior presumably through alterations of dopamine and serotonin metabolism.
Gabbay et al. (2009) [30]	12 suicidal adolescents, 18 non-suicidal adolescents with MDD, 15 controls	Cross-sectional case-control study	IFN-γ, TNF-α, IL-6, IL-1β, IL-4	Suicidal adolescents with MDD had significantly decreased plasma TNF-α concentrations compared to non-suicidal adolescents with MDD. IFN-γ was increased in both suicidal and non-suicidal adolescents with MDD compared to controls.	1. The cohort size was relatively modest; 2. a substantial proportion of patients was receiving psychotropic medications, which have been reported to induce negative immunoregulatory effects in adults with MDD; 3. due to the small sample, a multiple comparison correction to preserve statistical power was not carried out.	These preliminary findings suggest that immune system dysregulation may be associated with suicidal symptomatology in adolescent MDD.
Tonelli et al. (2008) [46]	Post-mortem samples from the Brodman area 11 of 34 completed suicides and 17 controls	Post-mortem study	The expression of mRNA species for TNF-α, IL-1β, IL-4, IL-5, IL-6, and IL-13 measured using real-time- CRP	Female suicide victims showed increased expression of IL-4 whereas males suicide victims exhibited increased IL-13. Female suicide victims also showed higher but not significant TNF-α expression.	Controls were not matched for age, toxicology was incomplete in 82% of cases, and limited information were available on psychological diagnosis. In addition, accurate interactions between alcohol on inflammatory processes with the expression of cytokines were not reported.	Increased expression of mRNA transcripts of Th2 cytokines were found in the human orbitofrontal cortex of completed suicides.
Kim et al. (2008) [31]	36 MDD patients with recent SA, 33 non-suicidal MDD patients, and 40 normal controls	Cross-sectional case-control study	IL-6, IL-2, IFN-γ, IL-4, TGF-β1, Th1/Th2 ratio	Non-suicidal MDD patients had higher IL-6 levels than suicidal MDD patients and normal controls, while suicidal MDD patients had lower IL-2 than non-suicidal patients and normal controls. Both MDD groups, with or without attempted suicide, had lower IFN-γ and IL-4 and higher TGF-β1 levels. HDRS scores had positive correlations with IL-6, IFN-γ, Th1/Th2 ratio and negative correlations with IL-4 in non-suicidal depression patients. Suicidal MDD patients had no significant correlations between the LSARS or RRR scores and cytokine release.	1. The effects of various confounding factors from these data cannot be excluded; 2. the existence of potential differences between those who agreed to participate and those that did not; 3. only in vitro mitogen-stimulated cytokine production (and not in vivo serum or plasma levels of cytokines) before the MDD treatment was measured.	The immune response has distinct differences between non-suicidal patients and suicidal patients.
Lee and Kim (2006) [32]	48 suicidal MDD patients, 47 non-suicidal MDD patients, 91 controls	Cross-sectional case-control study	In vitroTGF-β1 levels were investigated	In vitro TGF-β1 levels were significantly higher in suicidal MDD patients and non-suicidal MDD patients than controls.	1. In vitro but not CSF TGF-β1 levels were measured; 2. methods of suicide attempt were not controlled; 3. the fact that blood for the quantification of the TGF was drawn after fasting for controls and 2 h later from the admission for suicidal depressive patients may represent a bias.	In vitro TGF-β1 levels may play a relevant role in MDD but not in suicidal behavior.
Mendlovic et al. (1999) [36]	9 patients with MDD that lasted 2–12 weeks and 9 age- and sex- matched controls	Cross-sectional case-control study	IFN-γ, IL-2, IL-4, IL-5, IL-10 secretion measured from PHA-stimulated lymphocytes	The stimulated lymphocytes of suicidal depressed patients secreted significantly more IFN-γ than those of healthy controls. Non-suicidal depressed patients secreted significantly less IFN-γ as compared with controls. Also, suicidal depressed patients secreted less IL- 4 and IL-5 as compared with non-suicidal depressed patients (although the difference was not statistically significant).	The small sample size limits the generalization of the present findings	Th1 activation in suicidal depression might reflect a unique form of autoimmune suicide.

**Note:** 5-HIAA = 5-hydroxyindoleacetic acid; BMI = body mass index; CCL5, RANTES = chemokine (C-C motif) ligand 5, regulated on activation, normal T cell expressed and secreted; CCL11 = eotaxin-1; CNS = central nervous system; CRP = c-Reactive protein; CSF = cerebrospinal fluid; dlPFC = dorsolateral prefrontal cortex; ESE = explicit social exclusion; ESR = erythrocyte sedimentation rate; HDRS = Hamilton Depression Rating Scale; INC = social inclusion conditions; GR = glucocorticoid; HCC = hair cortisol concentration; HPA = hypothalamic pituitary adrenal; HVA = homovallinic acid; KYN = plasma kynurenine; IBA1 = ionized calcium binding adaptor molecule 1; IBA1-IR = IBA1-immunoreactive; ICAM-1 = intercellular adhesion molecule 1; IFN = interferon; IL = interleukine; IL-1ra = interleukine-1 receptor antagonist; LSARS = Lethality Suicide Attempt Rating Scale; MADRS = Montgomery-Asberg Depression Rating Scale; MCP-1, CCL2 = monocyte chemoattractant protein-1, chemokine (C-C motif) ligand 2; MHPG = 3-methoxy-4-hydroxy-phenylglycol; mRNA = messenger ribonucleic acid; MDD = major depressive disorder; MDD-M = major depressive disorder-melancholic; PHA = phytohaemagglutinin; RRR = Risk-Rescue Rating; SA = suicide attempt; SI = suicidal ideation; TGF = transforming growth factor; Th1 = T helper 1; Th2 = T helper 2; TNF = tumor necrosis factor; TRP = tryptophan; VEGF = vascular endothelial growth factor.

**Table 2:** Most relevant longitudinal studies showing the association between inflammatory cytokines and suicidal behavior.

Author (s), Year	Sample Characteristics	Study Design	Inflammation Measurement	Main Findings	Limitations	Conclusions
Bay-Richter et al. (2014) [28]	30 subjects (43% MDD) with SA; 36 healthy controls	2-year longitudinal study	IL-6; CSF kynurenic acid; CSF quinolinic acid	Quinolinic acid was increased and kynurenic acid decreased over time in suicidal patients *vs.* healthy controls. A significant association between lower kynurenic acid and severe depressive symptoms as well as a link between higher IL-6 levels and more severe suicidal symptoms were reported.	1. The study is correlational in nature and does not test direct causality in the patients; 2. Most patients were on psychoactive medications; 3. gender and age of both controls and patients were not matched; 4. repeated lumbar punctures are carried out only on suicide attempters and not healthy controls	A long-term dysregulation of the kyn pathway in the CNS of SA was demonstrated. An increased load of inflammatory cytokines was coupled to more severe symptoms.
Li et al. (2013) [49]	64 first-episode drug-naïve MDD patients and 64 matched healthy controls	8-week case-control study	TNF-α	Plasma TNF-α levels were significantly decreased after venlafaxine treatment. Compared to non-responders, responders had a greater reduction in TNF-α levels which was linked to the greater reduction rate of HDRS-17. The plasma TNF-α levels were equally higher in both suicidal and non-suicidal MDD patients relative to healthy controls on admission.	1. The naturalistic observation design may not completely control confounding factors such as selective bias; 2. the levels of serotonin and norepinephrine were not detected; 3. the dexamethasone/CRH test was not performed; 4. the long-term effects of venlafaxine on plasma TNF-α levels have been not evaluated.	MDD but not per se suicide was associated with the increased plasma TNF-α levels that may be inhibited using venlafaxine
O’Donovan et al. (2013) [37]	76 MDD and 48 healthy controls	Follow-up study	TNF-α; IL-6; IL-10; CRP	Patients with MDD and higher SI had higher inflammatory index scores than both controls and MDD patients with lower SI. Patients with lower SI were not different from controls on the inflammatory index as well. Follow-up analyses indicated that differences between MDD patients with high *vs.* lower SI were independent of depression severity and recent SA.	1. The cross-sectional study design; 2. The small sample size; 3. The assumption of antidepressants and other medications; 4. the use of a structured interview measure of depression severity (HDRS) and not self-report measure; 5. The inclusion of a small number of patients with SA in the previous month.	SI may be uniquely linked to inflammation in depressed patients.
Janelidze et al. (2013) [40]	41 patients with SA, 22 non-suicidal psychiatric patients, 43 healthy controls	12 year follow-up study	CCL11; IP-10; CXCL10; MIP-1β, CCL4; MCP-1, CCL2; MCP-4,CCL13; TARC,CCL17	CSF eotaxin-1, MIP-1β (CCL4), MCP-1 (CCL2), MCP-4 (CCL13), and TARC (CCL17) were significantly lower in SA. Lower chemokine levels were specifically linked to psychotic symptoms and pain.	1. The mismatch in gender distribution in the CSF Index study; 2. differences in the recruitment time between suicide attempter and healthy control groups in the CSF Index study; 3. multiple analyses were performed on the present data.	When compared to non-attempters, abnormally reduced chemokine levels in suicide attempters, both in the acute suicidal setting and after the long-term period, were reported.
Isung et al. (2012a) [42]	58 suicide attempters	13 year longitudinal study	IL-1α; IL-1β; IL-2; IL-4; IL-6; IL-8; IL-10; IFN-γ; TNF-α; MCP-1; EGF; VEGF	Significantly lower VEGF levels were found in the seven patients after a mean follow-up of 13 years before completing suicide. VEGF also showed a trend for negative correlation with the planning subscale of SIS. A trend might be shown for lower IL-2 and higher IFN-γ levels in suicide victims.	1. Degradation of several cytokines has been observed after 4 years of storage at –80 °C; 2. the data on smoking habits and exercise were not available.	Further support for a role of inflammation in the pathophysiology of suicidal behavior has been provided. VEGF may be significantly related with suicide risk.
Nassberger and Trask- man- Bendz (1993) [44]	Medication-free suicide attempters with depressive disorders	Follow-up study	Urine and CSF soluble IL-2 receptor concentration presumably reflecting an activation of T-lymphocytes	A median soluble IL-2 receptor concentration far above the range of healthy controls was found. High levels of the soluble IL-2 receptor concentration were also reported at follow-up. There was a tendency of an association between soluble IL-2 receptor concentration and the ratio of norepinephrine-epinephrine in 24-h urine, as well as plasma and CSF 4-hydroxy-3- methoxymethyl-glycol.	1. The small sample size limits the generalization of the present findings; 2. the study lacks of a placebo group.	Psychiatric patients investigated after a suicide attempt showed an imbalance of the immune system.

**Note:** CCL11 = eotaxin-1; COBY = course and outcome of bipolar youth; CXCL10 = C-X-C motif chemokine 10; CNS = central nervous system; CRH = corticotropin releasing hormone; CRP = c-Reactive protein; CSF = cerebrospinal fluid; EGF = epidermal growth factor; HDRS = Hamilton Depression Rating Scale; IFN-γ= interferon gamma; IL = interleukine; IP-10 = interferon gamma-induced protein 10; KYN = kynurenine; MCP-1, CCL2 = monocyte chemoattractant protein-1, chemokine (C-C motif) ligand 2; MCP-4, CCL13 = monocyte chemoattractant protein 4, C-C motif chemokine ligand 13; MDD = major deprerssive disorder; MIP-1b, CCL4 = macrophage inflammatory protein, chemokine (C-C motif) ligands 4; SA = suicide attempts; SI = suicide ideation; SIS = Suicide Intent Scale; TARC, CCL17 = thymus and activation regulated chemokine (C-C motif) ligand; TNF = tumor necrosis factor; VEGF = vascular endothelial growth factor.
